# Developmental and epileptic encephalopathies with germline *PIGA* variants in five Chinese children: a case report and literature review

**DOI:** 10.3389/fgene.2026.1770784

**Published:** 2026-03-27

**Authors:** Danping Huang, Min Liu, Weihao Ling, Xuejun Shao, Xuqin Chen

**Affiliations:** 1 Children’s Hospital of Soochow University, Suzhou, China; 2 Children’s Hospital of Shanghai, Shanghai, China

**Keywords:** case report, developmental and epileptic encephalopathies, epilepsy, multiple congenital anomalies-hypotonia-seizures syndrome 2, next-generation sequencing, *PIGA*, variant

## Abstract

**Background:**

Diseases associated with the germline *PIGA* gene include multiple congenital anomalies-hypotonia-seizures syndrome 2 (MCAHS2) and neurodevelopmental disorder with epilepsy. The clinical heterogeneity of *PIGA*-related diseases is extensive, so its diagnosis and treatment remain challenging.

**Methods:**

We identified five germline missense pathogenic/likely pathogenic variants in *PIGA* across six unrelated families (NM_002641.3, c.130C>T p.P44S, c.368C>T p.T123M, c.241C>T p.R81C, c.751T>C p.C251R, and c.985G>T p.V329L), of which three variants have not been reported previously.

**Results:**

In all probands, the clinical symptoms included early-onset epilepsy, hypotonia, dysmorphic features, and variable congenital anomalies. A literature review of 107 cases supports reclassification of the phenotypic spectrum into severe (15.9%), intermediate (72.0%), and milder (12.1%) categories. Notably, the phenotypes of the five cases were classified as severe (n = 2) or milder (n = 3), consistent with prior reports, but revealed population-specific traits such as universal febrile sensitivity and normal serum alkaline phosphatase levels that are in contrast to elevated levels often noted in Western cohorts. The three children with the milder phenotype were found to have pathogenic/likely pathogenic variants located in exon 2, while the two severe phenotypes showed these variants located in exons 3 and 5.

**Conclusion:**

Overall, we report three novel pathogenic/likely pathogenic variants that expand clinicians’ understanding of the genetic diversity within this phenotypic spectrum. These insights are expected to be valuable for future pathogenic/likely pathogenic variant analysis and accurate classification of clinical subtypes, which would help improve the understanding of *PIGA*-related diseases. In addition, our research contributes to ongoing efforts to elucidate the underlying molecular mechanisms and inform precision medicine approaches for affected individuals.

## Introduction

Developmental and epileptic encephalopathies (DEEs) (Specchio and Curatolo, 2021) are a group of severe early-onset epilepsies characterized by refractory seizures, developmental delays (DDs), or regression associated with persistent epileptic activities that often result in poor prognosis. Genetic variations are commonly responsible for the pathogenesis of DEEs, and there has been significant progress in the identification of genetic variations associated with encephalopathies in DEEs owing to the widespread use of molecular diagnostic techniques such as next-generation sequencing. The PIGA protein plays a crucial role in the production of glycophosphatidylinositol (GPI) anchor though a series of steps (Kinoshita and Fujita, 2016; Bellai-Dussault et al., 2019). Specifically, it is involved in the initial step leading to the production of an intermediate molecule called N-acetylglucosaminyl phosphatidylinositol or GlcNAc-PI (Watanabe et al., 1996; Watanabe et al., 1998). The GPI anchor, which is the ultimate product of the series of steps, is responsible for the attachment of numerous proteins to the cell membrane; these proteins are known as GPI-anchored proteins. The anchored proteins have diverse functions such as cell adhesion, signal transduction, and cellular protection. Somatic variants in the *PIGA* gene are the underlying cause of paroxysmal nocturnal hemoglobinuria (Brodsky, 2014), which manifests as episodes of blood in the urine (hemoglobinuria) due to the breakdown of red blood cells. Inherited (germline) variants in the *PIGA* gene can result in multiple congenital anomalies-hypotonia-seizures syndrome 2 (MCAHS2), which encompasses a spectrum of conditions exhibiting diverse features and varying degrees of severity (Johnston et al., 2012; Kato et al., 2014). Severely affected individuals typically exhibit profound intellectual developmental disorder (IDD), infantile spasms, hypotonia characterized by weak muscle tone, distinctive facial features, abnormalities in the central nervous system (brain and spinal cord), and other body systems (Cash et al., 2020). Individuals with milder manifestations are characterized by mild-to-moderate IDD and seizures that can typically be treated. Despite over 100 reported cases, the phenotypic spectrum remains incompletely characterized and comprises limited data from Asian populations. Prior classifications as “severe” or “milder” may oversimplify the continuum, and genotype–phenotype correlations regarding variant locations require refinement. Herein, we describe five Chinese cases with germline *PIGA* variants and emphasize the novel pathogenic/likely pathogenic variants, population-specific features, and insights into phenotypic reclassification and exon-specific associations.

## Materials and methods

Among the patients with DEEs evaluated at the Department of Neurology, Children’s Hospital of Soochow University, five were found to have germline *PIGA* gene variants. All cases were analyzed retrospectively, including epilepsy, development, electroencephalography (EEG) and magnetic resonance imaging (MRI) evaluations, genotype analysis, treatment, and evolution. This study was approved by the Ethics Committee of the Children’s Hospital of Soochow University. Written informed consent was obtained from the legal guardians of the patients to participate in our study.

### Sample collection

We collected EDTA-treated peripheral blood samples with the informed consent of the patients.

### DNA extraction

The peripheral blood genomic DNA samples of the trio families were extracted using the TIANGEN Blood Genome Magnetic Genomic DNA Kit (DP329-TA), according to manufacturer’s instructions. The extracted DNA samples were subjected to quality control using a Qubit 2.0 fluorimeter and electrophoresis with 0.8% agarose gel for further protocol.

### Whole-exome library construction

Protein-coding exome enrichment was performed using xGen Exome Research Panel v2.0 (IDT, IA, United States), which comprised 415,115 individually synthesized and quality-controlled probes; these target the 34 Mb protein-coding region (19,433 genes) of the human genome and encompass 39 Mb of the end-to-end tiled probe space.

### Sequencing

High-throughput sequencing was performed using the MGI NBSEQ-T7 sequencer that covered over 99% of the target sequences. The sequencing was performed at the Beijing Chigene Translational Medicine Research Center Co., Ltd., 100875, Beijing.

### Bioinformatics analysis

1. Quality control: The raw data were processed by fastq to remove adapters and filter low-quality reads.
2. Variant calling: The paired-end reads were performed using a Burrows–Wheeler aligner (BWA) to the ensemble the GRCh37/hg19 reference genome. The base quality score recalibration along with single-nucleotide polymorphism (SNP) and short indel calling were conducted using GATK. Based on the sequencing depth and variant quality, the SNPs and indels were screened as high quality to obtain reliable variants.
3. Variant annotation and pathogenicity prediction: The online system independently developed by Chigene (www.chigene.org) was used to annotate the database-based minor allele frequencies (MAFs) and obtain the pathogenicity of each gene variant based on the American College of Medical Genetics and Genomics (ACMG) practice guideline; the system also provides a serial software package for conservative analysis and protein product structure prediction. The databases for the MAF annotations include 1,000 genomes, dbSNP, ESP, ExAC, gnomAD, and Chigene in-house MAF database; furthermore, we used Provean, Sift, Polypen2_hdiv, Polypen2_hvar, pathogenic/likely pathogenic variant taster, MCap, CADD, and Revel software packages to predict the protein product structure variations. As a prioritized pathogenicity annotation to the ACMG guidelines, we used the OMIM, HGMD, and ClinVar databases as conferences of the pathogenicity of each variant. To predict the functional changes to the variants at the splicing sites, we used MaxEntScan, dbscSNV, SpliceAI, and GTAG software packages.
4. Copy number variation (CNV) detection and annotation: We used CANOE, CNVnator, DeviCNV, and ExomeDepth to detect the CNVs from whole-exome sequencing (WES) data, and all CNVs were annotated to obtain additional information on the population frequencies and possible effects. The population frequencies for the CNVs were obtained from the Database of Genomic Variants. To assess the inclusion of any established dosage-sensitive genes or regions and the possible impacts on gene function, each CNV was evaluated against a select set of haplo-insufficient and triple-sensitivity genes as well as genomic regions obtained from ClinGen and Database of Chromosomal Imbalance and Phenotype in Humans Using Ensembl Resource (DECIPHER).

## Results

**FIGURE 1:**
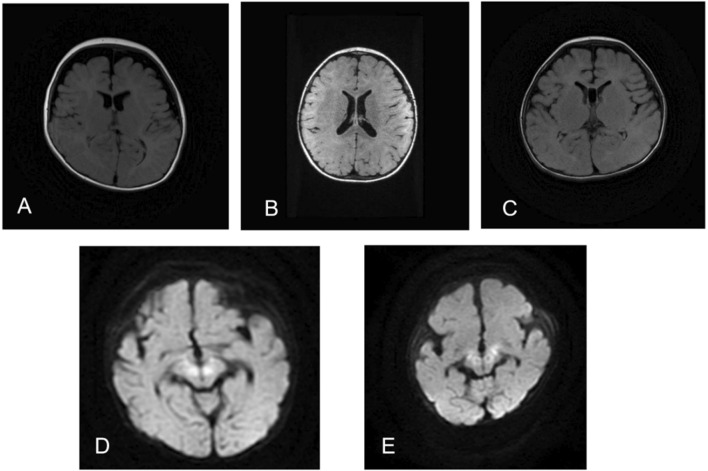
Magnetic resonance imaging (MRI) details of patients: **(A)** patient #1 at 8 months of age with widening of the extracerebral space (FLAIR); **(B)** patient #2 at 1 year of age with normal MRI (FLAIR); **(C)** patient #3 at 1 year of age with widening of the hyaline septum (FLAIR); **(D)** patient #4 at 2 months of age with high diffusion-weighted imaging (DWI) signals in the brainstem and basal ganglia; **(E)** patient #5 at 1 month of age with high DWI signals in the brainstem and basal ganglia.

**FIGURE 2:**
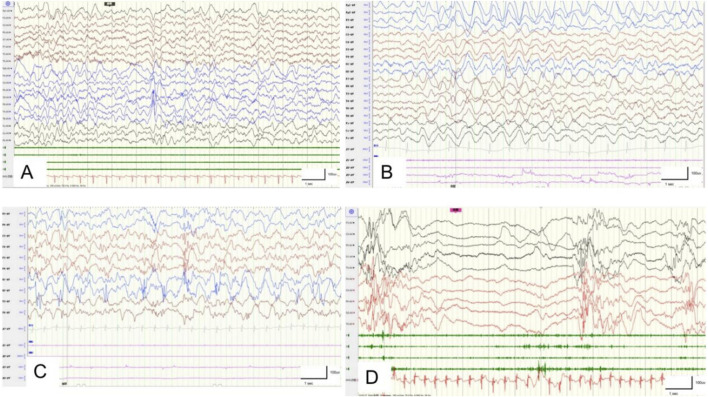
Electroencephalographic features of the patients: **(A)** patient #2 at 1 year of age with multifocal spike and slow waves; **(B)** patient #3 at 1 year of age with multifocal spike and slow waves; **(C)** patient #4 at 4 months of age with suppression burst features; **(D)** patient #5 at 2 months of age with suppression burst features. F, frontal; Fp, frontopolar; C, central; T, temporal; O, occipital; even numbers, right hemisphere; odd numbers, left hemisphere.

The clinical features of the different variants are summarized in Table 1, while the brain MRIs are shown in Figure 1 and EEG data are shown in Figure 2.

**TABLE 1:** Clinical summary of patients with the *PIGA* pathogenic/likely pathogenic variant.

Case	1	2	3	4	5
Mutation	c.130C>T (p.P44S)	c.368C>T (p.T123M)	c.241C>T (p.R81C)	c.751T>C (p.C251R)	c.985G>T (p.V329L)
Current age	5 years	2 years	2 years	Died	Died
Gestation	37 weeks	38 weeks	Full term	Full term	40 weeks
Sex	M	M	M	M	M
Facial dysmorphism	-	-	-	+	+
Joint contractures	-	-	-	+	+
Hypotonia	-	-	+	+	+
Hyperreflexia	-	-	-	+	+
Seizure onset	7 months	3 months	5 months	Neonate	Neonate
Seizure type	Tonic seizure	Focal seizure	Tonic seizure	Spasmodic seizures	Spasmodic seizures
EEG findings	Multifocal spike and slow waves	Multifocal spike and slow waves	Multifocal spike and slow waves	Suppression burst	Suppression burst
ASMs used	VPA and LEV	VPA, LEV, and LTG	VPA, LEV, and TPM	LEV and CZP	LEV and NZP
Seizure prognosis	Seizure-free at 4 years	Seizure-free at 1 year	Seizure-free at 1.5 years	Intractable	Intractable
Development	Global developmental delays, including both intelligence and motor skills	Global developmental delays, including both intelligence and motor skills	Global developmental delays, including both intelligence and motor skills	Early death	Early death
Restricted diffusion pattern	-	-	-	+	+
Elevated serum alkaline phosphatase	-	-	-	-	-

Abbreviations: M, male; +, present; -, absent; ASM, anti-seizure medication; LEV, levetiracetam; VPA, valproic acid; TPM, topiramate; LTG, lamotrigine; CZP, clonazepam; NZP, nitrazepam.

### Case 1

**FIGURE 3:**
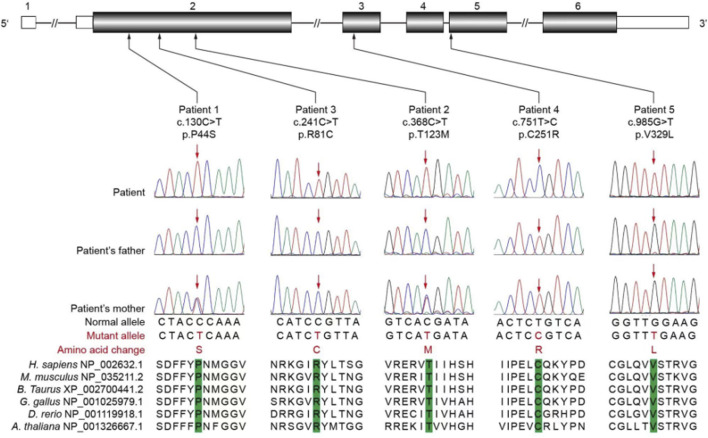
Schematic representation of the *PIGA* genomic structure, where the pathogenic/likely pathogenic variants are indicated based on the transcript variant 1 (GenBank accession number: NM_002641.3). The untranslated and coding regions are shown as white and black rectangles, respectively. All pathogenic/likely pathogenic variants occurred at evolutionarily conserved amino acids. The orthologous sequences were aligned using the CLUSTALW website.

The initial case involved a G2P2 boy born to a non-consanguineous family and delivered at 37 weeks with a birth weight of 2.5 kg. The mother of the boy and her ex-husband had a male child who experienced refractory epilepsy and died without undergoing genetic testing. At the age of 7 months, the boy began experiencing events characterized by tonic–clonic seizures with fever. The EEG data showed high amplitudes as well as sharp–slow and polyspike–slow wave complexes. The MRI showed a wide extracerebral space (Figure 1A). Meanwhile, the boy exhibited growth delays, including movement, language, and cognition. Although he can now walk independently, his motor coordination remains poor, while his language is limited to short sentences with impaired learning ability. The patient is currently 5 years old and has been free of seizures for 1.5 years under treatment with valproic acid (21 mg/kg/d) and levetiracetam (42 mg/kg/d). A novel variant c.130C>T (p.P44S) of the *PIGA* gene was identified in this patient through WES (Figure 3), and this was found to be inherited from his mother. It was rated to be a variant of uncertain significance (VUS) according to the ACMG guideline.

### Case 2

The second case was a G2P2 boy born to a non-consanguineous family and delivered at 38 weeks of gestation with a birth weight of 3.1 kg. He first experienced seizures at the age of 3 months after receiving the meningitis vaccine. The EEG data showed high-amplitude sharp-slow waves in the right frontal-parietal-temporal regions (Figure 2A), while the MRI was normal (Figure 1B). The patient was diagnosed with epilepsy and treated with valproic acid (38 mg/kg/d), levetiracetam (43 mg/kg/d), and lamotrigine (0.6 mg/kg/d). The boy has been free of seizures for 1 year now. At his current age of 2 years, the boy still demonstrates DDs in both motor and language skills, being unable to walk independently or speak fluently owing to his poor comprehension ability. A variant c.368C>T (p.T123M) was identified in the *PIGA* gene through WES (Figure 3); this was found to be inherited from his mother and classified as VUS according to the ACMG guideline.

### Case 3

The third case is a 2-year-old G1P1 boy born into a non-consanguineous family and delivered vaginally at full term. The boy experienced onset of seizures at the age of 5 months. The EEG data showed high-amplitude 1.5–2 Hz periodic waves (Figure 2B), while the MRI showed widened septum pellucidum (Figure 1C). The patient has been free of seizures for 9 months but shows DDs in both motor and language skills, along with below-normal muscle tone. He remains unable to walk or speak fluently despite treatment with valproic acid (24 mg/kg/d), levetiracetam (50 mg/kg/d), and topiramate (2.5 mg/kg/d). However, he still experiences occasional febrile convulsions. A *de novo* variant c.241C>T (p.R81C) of the *PIGA* gene (Figure 3) was identified in this patient via WES and was assessed as likely pathogenic.

### Case 4

The fourth case is a G1P1 boy born to a non-consanguineous family and delivered at full term under cesarean section. He first experienced seizures in the neonatal period, which manifested as a spasmodic seizure. The EEG data showed suppression-burst (Figure 2C) features, while the MRI revealed high diffusion-weighted imaging (DWI) signals (Figure 1D). His seizures were difficult to control and were accompanied by pronounced opisthotonus and hypertonia. He exhibited facial dysmorphisms, which included a depressed nasal bridge, short anteverted nose, and downturned corners of the mouth. In addition, he presented with gastrointestinal malformations, such as Hirschsprung disease, and had recurrent lung infections. He was treated with levetiracetam (36 mg/kg/d) and clonazepam (0.11 mg/kg/d). Unfortunately, he succumbed at the age of 18 months owing to these complications. A *de novo* variant c.751T>C (p.C251R) of the *PIGA* gene (Figure 3) was identified in the patient via WES, which was rated to be likely pathogenic according to ACMG guidelines.

### Case 5

In the last case, a G5P4 boy was born to a non-consanguineous family at 40+2 weeks with a birth weight of 3.6 kg. There was no reported history of epilepsy in his family. The onset of seizures occurred when the boy was 20 days old. The EEG data showed suppression-burst (Figure 2D) features, while the MRI revealed high DWI signals (Figure 1E). Additionally, the boy presented multiple deformities, including facial deformities, hearing abnormalities, and atrial septal defects. He was treated with levetiracetam (55 mg/kg/d) and nitrazepam (0.2 mg/kg/d). During hospital discharge, the seizure frequency ranged from once every 4–5 d up to more than once per day over time. Unfortunately, he succumbed before reaching 1 year of age owing to these complications. The WES showed a *de novo* variant c.985G>T (p.V329L) (Figure 3), which was rated to be likely pathogenic according to the ACMG guidelines.

### Genetic analysis

The samples from the five families were detected by WES and verified through Sanger sequencing, and a total of five missense variants were found in the *PIGA* gene (Figure 3). Among these variants, two have been reported previously (Cash et al., 2020; Bayat et al., 2020; Jiao et al., 2020), whereas three variants were novel and have not been reported internationally. These three novel variants were assessed as two likely pathogenic and one VUS variants according to ACMG guidelines (Seo et al., 2020).

The c.130C>T (p.P44S) variant has not been reported in the literature or documented in the ClinVar database. This missense pathogenic/likely pathogenic variant was maternally inherited; it is not included in the normal population database (PM2_Supporting) and is predicted to be highly damaging to the protein structure by SIFT, PolyPhen-2, and MutationTaster (PP3). Based on available evidence, this variant is defined as VUS (PM2_Supporting + PP3). The c.751T>C (p.C251R) variant has not been reported in the literature. As a *de novo* variant (PS2), it is not included in the normal population database (PM2_Supporting) and is predicted to be highly damaging to the protein structure by SIFT, PolyPhen-2, and PP3. The variant was defined as likely pathogenic (PS2 + PM2_Supporting + PP3). The c.985G>T (p.V329L) variant has not been reported in relevant literature, and there is a VUS report in the ClinVar database; it is also a *de novo* variant (PS2) that is not included in the normal population database (PM2_Supporting) and is predicted to be highly damaging to the protein structure by SIFT, PolyPhen-2, and PP3. Moreover, variants of the same amino acid site, c.986T>C (p.V329A) (Seo et al., 2020), have been reported to be pathogenic (PM5). The variant was rated to be likely pathogenic (PS2 + PM2_Supporting + PM5 + PP3). The five variants that we found were all missense pathogenic/likely pathogenic variants. In the two children with MCAHS2, c.751T>C (p.C251R) was located in exon 3, while c.985G>T (p.V329L) was located in exon 5. The other three children with milder clinical phenotypes had pathogenic/likely pathogenic variants in exon 2.

## Literature review

Based on the phenotype differences observed in the patients, all reported cases of *PIGA* variants were reviewed. Thus, a total of 107 cases (Johnston et al., 2012; Kato et al., 2014; Cash et al., 2020; Bayat et al., 2020; Jiao et al., 2020; Swoboda et al., 2014; Tarailo-Graovac et al., 2015; Joshi et al., 2016; Lin et al., 2018; Xie et al., 2018; Yang et al., 2018; Cabasson et al., 2020; Neuhofer et al., 2020; Perk Yucel and Mutlu Albayrak, 2020; Bayat et al., 2021; Flores-Torres et al., 2021; Liu et al., 2022; Salinas-Marín et al., 2022; Bayat et al., 2023; Crenshaw et al., 2023; Ikeda et al., 2023) were analyzed together with our cases. In the described cases, the median age at which the patient initially presented with clinical manifestations of specific organs or systems owing to the *PIGA* variant was 6 months (range, neonatal period to 5 years). Of these, 29% of the patients eventually died (range: 15 d to 48 years), with a minimum age of death of 15 d and an average age of death of 5.6 years.

**FIGURE 4:**
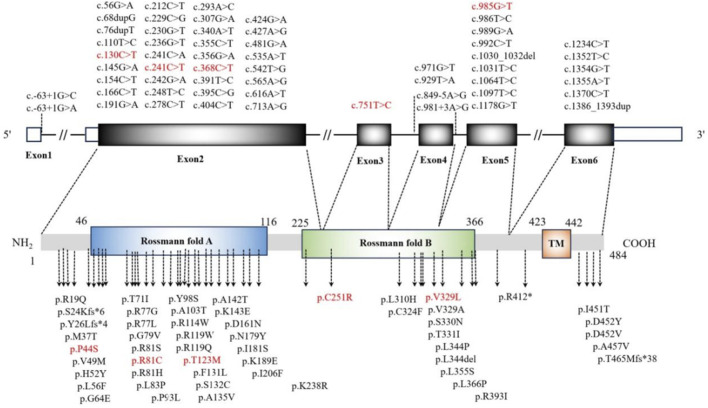
Positions of the pathogenic variants in the *PIGA* gene and PIGA protein. The known pathogenic variants are shown in black, while the variants of the cases in this study are shown in red.

The phenotypic spectrum of the *PIGA* variants included epileptic seizures, profound DDs, IDD, multiple congenital malformations, and early death. Neurological manifestations such as DDs/regression, hypotonia, and epilepsy were the main characteristic features of patients with the *PIGA* variant. In general, the phenotypic spectrum caused by the pathogenic/likely pathogenic variants of the *PIGA* germline was earlier classified into two as severe and less severe. However, we found that the phenotypes of the 107 patients reported to date could hardly be divided into these two groups. Of the 107 reported cases, the less severe group (mild-to-moderate IDD/DD, treatable epilepsy, lack of dysmorphic features) made up approximately 12.1% (13/107), while the severe group (profound IDD/DD, treatment-refractory epilepsy, dysmorphic features, multiorgan malformations, and early death) comprised approximately 15.9% (17/107) and medium group (between severe and less severe groups) accounted for 72.0% (77/107). The exon-specific features of these variants are listed in Table 2. To date, 56 different variant sites have been detected, where 48 are missense variants, 4 are splice-site variants, and 4 are truncating variants. As shown in Figure 4, more than half of the variants were located in the Rossmann fold A (34/56), while another hot spot for pathogenic variants was found in the C-terminal part of the second Rossmann fold B.

**TABLE 2:** Summary of key clinical features of patients with *PIGA* variants in different exons.

Clinical data % (n/n) or n	Exon 2 (n = 65)	Other exons (n = 42)	Total morbidity % (n/N)
Age of onset (months)	5 (0–13)	6 (neonatal-60)	​
Outcome	​	​	​
Alive	81.5% (53/65)	54.8% (23/42)	71.0% (76/107)
Dead	18.5% (12/65)	45.2% (19/42)	29.0% (31/107)
Abnormal development	​	​	​
Profound-to-severe	70.8% (46/65)	69% (29/42)	70.1% (75/107)
Mild-to-moderate	20.0% (13/65)	0% (0/42)	12.1% (13/107)
Epilepsy	​	​	​
Refractory epilepsy	56.9% (37/65)	57.1% (24/42)	57% (61/107)
Controlled	35.4% (23/65)	9.5% (4/42)	25.2% (27/107)

Data are shown as prevalence % (n/n) or n (number).

## Discussion

The pathogenic/likely pathogenic variants of the *PIGA* gene have been associated with a spectrum of diseases, displaying a wide range of phenotypic severity in affected children. These manifestations span from milder forms characterized by epilepsy and IDD without obvious structural malformations to more severe MCAHS2 characterized by early-onset epileptic encephalopathy, hypotonia, multiple congenital malformations, and early death (Bayat et al., 2020; Cabasson et al., 2020; Perk Yucel and Mutlu Albayrak, 2020). To date, there are 112 cases of individuals with the *PIGA* pathogenic/likely pathogenic variant published in medical literature. In this study, we focused on the germline variants of the *PIGA* gene in five Chinese patients with DEEs. Among them, the p.P44S, p.C251R, and p.V329L were novel variants that had not been reported previously.

Children with MCAHS2 may have multiple congenital malformations, including hypotonia, facial deformities, joint contractures, cardiovascular abnormalities, urinary system abnormalities, and digestive system abnormalities. Consistent with previous literature reports (Johnston et al., 2012; Kato et al., 2014; Cash et al., 2020; Bayat et al., 2020; Jiao et al., 2020), cases 4 and 5 in this study exhibited facial deformities and multisystem abnormalities. Consistent with the genotype–phenotype heterogeneity, there was heterogeneity in the MRI data. Several authors (Kato et al., 2014; Cash et al., 2020; Bayat et al., 2020) reported restricted diffusion on the brainstem, basal ganglia, and thalamus, similar to cases 4 and 5. Other cases have also reported widening of the transparent septum and widening of the extracerebral space (Cabasson et al., 2020). Additionally, elevated alkaline phosphatase (ALP) has been proposed as a diagnostic marker (Johnston et al., 2012; Bayat et al., 2020). In our Chinese cohort, all cases exhibited early-onset epilepsy, hypotonia, dysmorphisms, and anomalies, aligning with typical phenotypes but showing certain distinctions: universal febrile sensitivity (not universally reported previously) and normal ALP (in contrast with elevations in ∼15% of Western cohorts; Johnston et al., 2012; Bayat et al., 2020).

Epilepsy is the most common feature in the *PIGA* gene pathogenic/likely pathogenic variant-related diseases (Bayat et al., 2020). All reported cases related to the *PIGA* gene variants have exhibited seizures to date, with the age of onset ranging from neonatal to infancy and seizures often being refractory (Johnston et al., 2012). The seizure types include myoclonic, tonic, focal, and spasticity seizures. All of our patients experienced seizures during infancy, and two of the patients had neonatal epilepsy with burst suppression. Furthermore, all children in our study had heat-sensitive seizures. Treatment is a challenging task for controlling recurrent and persistent seizures. Valproic acid, levetiracetam, and topiramate are the most commonly used drugs in our study. A recent report by Joshi et al. (2016) highlighted the efficacy of the ketogenic diet for *PIGA* gene variant-related diseases, but other authors (Xie et al., 2018; Cabasson et al., 2020) have suggested the opposite. It has been reported that seizures in children with the pathogenic/likely pathogenic variants of the *PIGA* gene are caused by the deficiency of pyridoxal phosphate (PLP) (Chiyonobu et al., 2014); these *PIGA* gene variants affect the biosynthesis of GPI or process of protein attachment, leading to the inability of PLP to participate in the synthesis of the inhibitory gamma-aminobutyric acid. Notably, the targeted drugs butyrate and pyridoxine have been reported to be effective in the treatment of seizures in patients with *PIGM* or *PIGO* pathogenic/likely pathogenic variants, respectively (Krawitz et al., 2012). Based on consideration of etiological treatment, some researchers (Kato et al., 2014; Lin et al., 2018) attempted to use high-dose pyridoxine to treat patients with *PIGA* pathogenic/likely pathogenic variants, but the results were not significantly effective. At present, there is no specific treatment, and there may be important breakthroughs in future therapeutic interventions with the deepening of research on the pathological mechanisms of *PIGA* gene variability.

The gene and protein positions of the five cases are depicted in Figure 3. It is well known that *PIGA*-related diseases exhibit diverse clinical features and varying degrees of severity (Johnston et al., 2012; Cash et al., 2020). The affected individuals have different phenotypes ranging from severe to less severe. Severe symptoms typically manifest as profound IDD/DD, refractory epilepsy, weak muscle tone (hypotonia), distinctive facial features, multisystem deformities, and premature death, whereas those with milder presentations generally display mild-to-moderate IDD/DD and treatable seizures. The fact that two of the five patients in the present report exhibited a severe phenotype while the remaining three cases had a milder phenotype probably suggests the lineage characteristics of the *PIGA* gene variant-related diseases.

Bayat et al. (2020) found that the majority of the *PIGA* variants are located in the Rossmann fold A, while another hot spot for the *PIGA* variants was found in the C-terminal part of the second Rossmann fold B. The pathogenic/likely pathogenic variants in all five children in our study occurred in the hot spots. Two variants were reported to be milder (p.Thr123Met) (Bayat et al., 2020; Jiao et al., 2020) and variable (p.Arg81Cys) (Cash et al., 2020). The novel variants p.Pro44Ser (VUS), p.Cys251Arg, and p.Val329Leu (likely pathogenic) expand the spectrum; p.Val329Leu adjoins pathogenic p.Val329Ala (Seo et al., 2020). In this study, a genotype–phenotype correlation was identified: the milder phenotypes were associated with exon 2 variants (cytoplasmic domain), while the severe phenotypes were linked to exons 3/5 variants. The literature supports the milder outcomes for the exon 2 variants (Bayat et al., 2020; Swoboda et al., 2014; Liu et al., 2022; Bayat et al., 2023). Table 2 shows higher rates of controllable epilepsy (70% vs. 40%) and lower rates of severe DDs (50% vs. 80%) in patients with exon 2 variants. Among the 65 reported cases with exon 2 variants, most presented with milder or intermediate phenotypes, suggesting residual protein functions. The variants in exons 3/5 may disrupt critical domains, yielding severe phenotypes (Cash et al., 2020; Bayat et al., 2020; Bayat et al., 2021; Salinas-Marín et al., 2022). This phenotypic gradient refines the genotype–phenotype correlations to aid prognosis and classification. The reason why variants in exon 2 probably result in relatively mild clinical phenotypes is still unknown. As the interactions and functional and structural roles of the PIGA protein remain unclear, the impact of this domain remains to be explored. Thus, more patients need to be identified to improve the associations between these variants and phenotypes.

## Conclusion

In summary, germline *PIGA* variants result in a continuous phenotypic spectrum from milder IDD/DD and treatable epilepsy to severe MCAHS2. This X-linked disorder informs prenatal testing for subsequent pregnancies. Our report of five Chinese cases, including three novel variants, expands the genetic diversity and reveals population-specific features. The exon location influences the severity of MCAHS2, where exon 2 is linked to milder outcomes and aids early identification and subtype classification of MCAHS2. Thus, further studies can help elucidate the disease mechanisms and advance precision medicine.

## Data Availability

The original contributions presented in the study are publicly available. This data can be found in the Clinvar repository, with the accession number numbers: SCV007334756, SCV007334758, SCV007334759, SCV007334760, SCV007334761.
